# Supplementary material for: A Likelihood Approach for Real-Time Calibration of Stochastic Compartmental Epidemic Models
Source: PLoS Comput Biol. 2017 Jan 17;13(1):e1005257. doi: 10.1371/journal.pcbi.1005257 (PMC5240920; doi:10.1371/journal.pcbi.1005257)
Supplement: S1 File — (TAR.GZ) [file pcbi.1005257.s014.tar.gz › HSPH_Online-SI-Revision/Additions/IPoi10/n100-extreme_noisy/n100-extreme_noisy_BENCH_table-8.pdf]

1	R0	{2.76052, 16.3913}	{2.44077, 12.7635}	{{2.15962, 10.7214}, {2.688, 14.8279}}	{11.5831, 22.1326}	inf	695.	488.224	{106., 1124.}	60.3994	0.401899	newly	289.367	405.948	{79., 844.}	85.0397	0.30657	Reff	2.73137	2.41551	{2.14608, 2.65985}	11.564	ar	92090.	87674.1	{83312., 91405.}	4.79523
2	R0	{2.18202, 15.1734}	{2.66398, 18.3266}	{{2.31344, 14.661}, {2.81635, 19.898}}	{22.0877, 21.7621}	inf	2011.	7448.95	{786., 17100.}	290.178	0.53103	newly	1160.	3512.57	{465., 7192.}	224.118	0.477691	Reff	2.10076	2.30951	{1.83026, 2.70406}	16.1177	ar	84264.	90821.8	{86707., 92752.}	7.78247
3	R0	{2.57891, 12.5971}	{2.85676, 18.3459}	{{2.86501, 18.4249}, {2.86501, 18.4249}}	{10.9699, 45.6362}	inf	340.	252.419	{21., 600.}	67.3909	0.499378	newly	303.	139.935	{13., 309.}	65.5275	0.648714	Reff	2.5648	2.84729	{2.83808, 2.86386}	11.2026	ar	90302.	92983.2	{92871., 93196.}	3.02206
4	R0	{2.5856, 9.28508}	{1.47512, 14.5589}	{{1.0158, 8.09734}, {1.97176, 19.5412}}	{42.9487, 61.6707}	inf	8.	2.55	{0., 6.}	72.2917	0.820981	newly	20.	1.66667	{0., 4.}	91.6667	1.48546	Reff	2.58508	1.53504	{1.08884, 1.97174}	40.6193	ar	90312.	26632.3	{1., 75178.}	70.5108
5	R0	{2.4406, 12.1341}	{1.67901, 14.7864}	{{1.13227, 8.84894}, {2.22641, 19.6041}}	{31.8618, 30.7577}	inf	184.	25.2041	{2., 70.}	87.5444	1.28399	newly	170.	14.7347	{0., 43.}	91.3325	1.56337	Reff	2.43279	1.78588	{1.30773, 2.31977}	27.9534	ar	88597.	59420.7	{14., 86655.}	33.3458
6	R0	{2.72155, 15.4094}	{2.44658, 15.8164}	{{2.32014, 14.7828}, {2.76849, 18.33}}	{10.8019, 7.74601}	inf	411.	550.548	{52., 1458.}	94.6864	0.408625	newly	302.	330.306	{31., 846.}	79.4115	0.421825	Reff	2.7028	2.43873	{2.29151, 2.75971}	10.5389	ar	91682.	88399.9	{86336., 92108.}	3.81784
7	R0	{2.63781, 14.3935}	{2.33194, 17.3991}	{{1.94617, 14.0718}, {2.43839, 18.743}}	{11.6, 25.2621}	inf	448.	242.525	{15., 609.}	65.538	0.597481	newly	307.	123.729	{11., 285.}	70.0105	0.718421	Reff	2.61787	2.36521	{2.32467, 2.44686}	9.65214	ar	91192.	87233.1	{87141., 88668.}	4.3413
8	R0	{2.82391, 16.104}	{2.75487, 18.037}	{{2.32014, 14.7828}, {2.86656, 18.9813}}	{3.71531, 14.7149}	inf	560.	326.422	{41., 727.}	58.2785	0.502486	newly	385.	178.328	{19., 384.}	59.3791	0.56776	Reff	2.79954	2.78305	{2.73253, 2.86309}	2.6944	ar	92664.	92374.4	{92065., 93184.}	0.769649
9	R0	{2.80645, 14.6457}	{1.85338, 19.095}	{{1.34361, 18.7523}, {2.1968, 19.9579}}	{33.9599, 31.266}	inf	226.	27.1569	{3., 49.}	87.9837	1.1774	newly	148.	12.4706	{1., 27.}	91.5739	1.43421	Reff	2.79573	1.94919	{1.34344, 2.19662}	30.2797	ar	92567.	70314.4	{46418., 84665.}	24.0394
10	R0	{1.86213, 5.4271}	{1.44284, 15.1428}	{{1.0158, 8.09734}, {1.85141, 19.7105}}	{25.9602, 179.022}	inf	32.	3.91803	{0., 8.}	87.7561	1.21064	newly	74.	2.2459	{0., 4.}	96.965	1.847	Reff	1.8607	1.47628	{1.07192, 1.85121}	25.2337	ar	75362.	25816.8	{2., 72317.}	67.6858
11	R0	{2.27349, 14.6338}	{2.84754, 18.2519}	{{2.86501, 18.4249}, {2.93183, 18.9272}}	{25.718, 26.1634}	inf	1278.	1587.88	{211., 3488.}	77.5396	0.379563	newly	843.	860.899	{122., 1840.}	63.9314	0.385484	Reff	2.22036	2.77504	{2.69706, 2.86166}	25.3481	ar	85805.	92710.7	{92833., 93515.}	8.24373
12	R0	{2.77261, 14.1894}	{1.26091, 14.7819}	{{1.18979, 13.6259}, {1.34361, 18.7523}}	{54.5226, 12.5788}	inf	206.	9.86111	{1., 22.}	95.2131	1.56174	newly	178.	5.44444	{1., 13.}	96.9413	1.79593	Reff	2.76385	1.28566	{1.18966, 1.3435}	53.483	ar	92508.	28483.7	{11., 46890.}	69.2095
13	R0	{2.65402, 19.3935}	{2.51509, 17.3964}	{{2.41197, 16.4137}, {2.59755, 18.2464}}	{5.61793, 10.4124}	inf	1354.	2191.91	{221., 4528.}	116.131	0.432208	newly	658.	1148.09	{119., 2527.}	123.94	0.428777	Reff	2.59651	2.41677	{2.21147, 2.53235}	7.05355	ar	91184.	89329.7	{87877., 90532.}	2.1093
14	R0	{1.75297, 4.45354}	{1.41159, 17.7734}	{{1.18979, 13.6259}, {1.70573, 19.5412}}	{23.1251, 299.084}	inf	18.	5.26087	{1., 10.}	77.5362	0.787488	newly	29.	2.3913	{0., 7.}	91.7541	1.54851	Reff	1.75187	1.45318	{1.22612, 2.19524}	22.7876	ar	71263.	36539.4	{6., 83794.}	52.8488
15	R0	{1.99928, 7.1501}	{1.4354, 16.2612}	{{1.04117, 12.1226}, {1.79789, 19.7105}}	{28.826, 127.426}	inf	97.	4.37778	{0., 10.}	95.4868	1.61884	newly	168.	2.06667	{0., 5.}	98.7698	2.28131	Reff	1.99548	1.50099	{1.10125, 1.91343}	26.1821	ar	79602.	39535.5	{3., 75275.}	50.6327
16	R0	{2.67398, 10.3134}	{1.35271, 18.2825}	{{1.22638, 16.005}, {1.36512, 19.5412}}	{49.4123, 78.1853}	inf	44.	4.67568	{1., 9.}	89.3735	1.17963	newly	54.	1.94595	{0., 5.}	96.3964	1.85234	Reff	2.67219	1.35753	{1.22377, 1.36508}	49.1978	ar	91289.	29200.4	{4., 48415.}	68.0133
17	R0	{2.44634, 16.3226}	{2.79332, 19.6663}	{{2.81635, 19.898}, {2.81635, 19.898}}	{14.2884, 20.8478}	inf	1288.	874.614	{74., 1822.}	61.0881	0.486785	newly	777.	428.457	{42., 933.}	59.9816	0.535947	Reff	2.39443	2.7594	{2.70144, 2.81246}	15.2851	ar	88710.	92465.6	{92436., 92833.}	4.28736
18	R0	{2.02168, 10.1676}	{2.25368, 15.1985}	{{2.22641, 14.8348}, {2.35866, 16.9062}}	{11.4757, 49.4801}	inf	354.	354.6	{31., 859.}	81.8832	0.50562	newly	351.	223.033	{19., 529.}	68.7844	0.590702	Reff	2.00733	2.22773	{2.18954, 2.22563}	10.9799	ar	80299.	83706.6	{84469., 85304.}	7.57623
19	R0	{2.89969, 15.009}	{1.60401, 18.6244}	{{1.22638, 15.4344}, {2.1968, 19.9579}}	{44.6832, 27.464}	inf	224.	14.075	{1., 32.}	93.7165	1.51092	newly	179.	6.375	{0., 18.}	96.4385	1.83462	Reff	2.88972	1.71559	{1.34326, 2.19656}	40.631	ar	93349.	51026.4	{7., 84341.}	45.3381
20	R0	{2.68933, 7.34928}	{1.57082, 15.7553}	{{1.14639, 10.2835}, {1.97176, 19.7105}}	{41.8519, 114.378}	inf	3.	2.61905	{0., 7.}	67.7249	0.445926	newly	7.	1.34921	{0., 5.}	81.6327	1.13828	Reff	2.68909	1.60021	{1.22633, 1.97174}	40.9073	ar	91574.	31501.6	{1., 75937.}	65.703
21	R0	{2.15352, 9.28074}	{1.64829, 15.7245}	{{1.23973, 12.378}, {2.22641, 19.7105}}	{26.4469, 69.9047}	inf	131.	13.5778	{1., 39.}	89.6353	1.33024	newly	151.	8.71111	{0., 26.}	94.2311	1.73377	Reff	2.14827	1.71467	{1.29998, 2.22554}	21.9712	ar	83845.	53874.1	{5., 84756.}	36.3436
22	R0	{2.21953, 9.04387}	{1.41806, 16.4513}	{{1.18979, 10.1673}, {1.66638, 19.7105}}	{36.1225, 83.6253}	inf	114.	4.37037	{1., 12.}	96.1663	1.70458	newly	136.	2.14815	{0., 7.}	98.4205	2.24964	Reff	2.21565	1.44617	{1.21715, 1.67365}	34.7461	ar	84856.	35518.1	{3., 67996.}	58.143
23	R0	{2.21987, 14.5347}	{2.22641, 14.8348}	{{2.22641, 14.8348}, {2.22641, 14.8348}}	{0.294612, 2.06471}	inf	1324.	1856.39	{244., 4806.}	95.2007	0.380371	newly	822.	1131.22	{150., 2878.}	91.4512	0.370844	Reff	2.16759	2.14874	{2.02329, 2.2157}	2.44775	ar	85067.	84936.8	{84679., 85305.}	0.249375
24	R0	{2.84268, 17.9659}	{1.9596, 14.2431}	{{1.94617, 14.0718}, {1.94617, 14.0718}}	{31.065, 21.3279}	inf	799.	517.151	{36., 1444.}	63.0977	0.534373	newly	439.	316.038	{28., 830.}	62.4962	0.492406	Reff	2.80769	1.95041	{1.89068, 1.94469}	30.5333	ar	92836.	78645.7	{77769., 78696.}	15.2853
25	R0	{2.52706, 10.3679}	{1.21904, 13.6541}	{{1.22638, 13.6259}, {1.22638, 13.6259}}	{51.7606, 33.0095}	inf	97.	9.51852	{1., 31.}	90.1871	1.2709	newly	97.	4.74074	{0., 16.}	95.1126	1.63145	Reff	2.52279	1.21553	{1.18969, 1.22625}	51.8178	ar	89623.	22612.9	{16., 36055.}	74.7689
26	R0	{2.62835, 19.1088}	{2.64484, 18.2199}	{{2.31344, 14.661}, {2.81635, 19.898}}	{7.47934, 8.84033}	inf	1699.	1481.1	{168., 3902.}	61.6397	0.396638	newly	814.	772.145	{83., 1917.}	63.7018	0.384161	Reff	2.55757	2.60223	{2.26673, 2.80263}	7.48158	ar	90908.	90902.8	{86465., 92612.}	2.34005
27	R0	{2.76831, 19.4155}	{2.47638, 14.3708}	{{2.45256, 14.1192}, {2.5458, 15.1849}}	{10.5454, 25.9827}	inf	2186.	3154.42	{341., 7019.}	98.4574	0.403944	newly	1084.	2081.95	{232., 4645.}	130.76	0.402627	Reff	2.67358	2.34039	{2.16125, 2.46325}	12.4621	ar	92175.	88924.6	{88496., 89732.}	3.52631
28	R0	{2.96609, 16.8903}	{2.89358, 15.0048}	{{2.44675, 11.6528}, {2.99911, 15.963}}	{3.76844, 11.1633}	inf	591.	298.565	{24., 660.}	64.1095	0.67083	newly	383.	222.984	{18., 478.}	64.3098	0.635408	Reff	2.94132	2.88399	{2.4443, 2.99524}	3.8186	ar	94024.	90101.5	{88470., 94161.}	4.25324
29	R0	{2.17046, 14.39}	{2.22641, 14.8348}	{{2.22641, 14.8348}, {2.22641, 14.8348}}	{2.57779, 3.09104}	inf	1169.	867.109	{125., 2599.}	65.8887	0.486325	newly	743.	537.674	{74., 1549.}	64.483	0.48182	Reff	2.12193	2.19106	{2.12066, 2.22009}	3.39989	ar	84001.	84951.4	{84655., 85239.}	1.13143
30	R0	{2.88772, 12.4647}	{1.53886, 14.5562}	{{1.0158, 8.09734}, {2.1968, 19.5412}}	{46.7103, 32.0049}	inf	43.	3.73239	{0., 11.}	91.32	1.55083	newly	59.	2.23944	{0., 6.}	96.2043	1.92298	Reff	2.88584	1.57311	{1.01578, 2.20877}	45.4888	ar	93273.	28968.7	{1., 84771.}	68.9421
31	R0	{2.46831, 8.14308}	{1.51232, 11.8866}	{{1.0158, 8.09734}, {2.45321, 18.9272}}	{40.7686, 50.5665}	inf	13.	1.44	{0., 4.}	88.9231	1.39634	newly	16.	1.24	{0., 4.}	92.25	1.60522	Reff	2.46792	1.51228	{1.01578, 2.45319}	40.7631	ar	88762.	16643.1	{1., 84106.}	81.6099
32	R0	{1.93236, 7.38987}	{2.63258, 16.2471}	{{2.22212, 13.7658}, {2.86501, 18.4249}}	{37.5943, 119.857}	inf	96.	30.6232	{4., 81.}	70.6371	0.803874	newly	158.	19.2609	{1., 52.}	87.8096	1.27086	Reff	1.92828	2.6408	{2.22181, 2.86469}	37.9442	ar	77654.	86022.8	{75888., 93120.}	20.3277
33	R0	{2.82251, 17.6954}	{2.5657, 14.4413}	{{2.48103, 13.7658}, {2.59564, 14.6975}}	{9.20155, 18.4694}	inf	586.	420.371	{20., 1057.}	65.0694	0.603089	newly	325.	294.065	{14., 750.}	69.201	0.551369	Reff	2.7977	2.55373	{2.47165, 2.59195}	8.83336	ar	92745.	87261.9	{88838., 90647.}	5.95594
34	R0	{1.87083, 12.3994}	{2.76175, 19.4895}	{{2.76795, 19.6041}, {2.81635, 19.898}}	{47.6214, 57.1809}	inf	1336.	2898.83	{467., 6858.}	150.232	0.392806	newly	975.	1365.15	{235., 3114.}	87.595	0.338106	Reff	1.81905	2.63074	{2.42011, 2.7566}	44.6217	ar	76282.	92066.5	{92020., 92721.}	20.6923
35	R0	{2.95779, 16.3003}	{2.14598, 15.0267}	{{1.75668, 13.8428}, {2.22641, 17.1868}}	{27.4465, 11.4272}	inf	233.	83.0727	{1., 219.}	69.1689	0.861268	newly	159.	53.1273	{1., 148.}	69.3082	0.916436	Reff	2.94682	2.19204	{1.86872, 2.22623}	25.6132	ar	93873.	80940.1	{75096., 85179.}	13.7771
36	R0	{2.18718, 14.261}	{2.5349, 14.2037}	{{2.48103, 13.7658}, {2.59564, 14.6975}}	{15.8979, 3.27895}	inf	1258.	4830.31	{642., 10771.}	304.959	0.526757	newly	857.	3135.88	{424., 6809.}	285.585	0.514138	Reff	2.13862	2.32406	{2.04231, 2.5024}	10.4061	ar	84379.	89731.2	{88879., 90561.}	6.34304
37	R0	{2.44912, 9.31118}	{1.38764, 15.2885}	{{1.0158, 8.09734}, {1.67375, 19.7105}}	{43.3412, 69.5076}	inf	41.	3.73585	{0., 13.}	90.8882	1.45333	newly	54.	2.26415	{0., 6.}	95.8071	1.82574	Reff	2.44728	1.40476	{1.01577, 1.67362}	42.5992	ar	88442.	21187.4	{2., 67937.}	76.0437
38	R0	{2.05949, 5.72514}	{1.41557, 15.624}	{{1.0158, 8.09734}, {1.85141, 19.5412}}	{31.4965, 173.578}	inf	11.	3.12963	{1., 6.}	71.5488	0.733047	newly	26.	1.25926	{0., 3.}	95.1567	1.70293	Reff	2.05842	1.4737	{1.10129, 1.869}	28.8363	ar	81587.	27941.	{2., 74426.}	65.9399
39	R0	{1.75505, 8.52404}	{2.80326, 19.4401}	{{2.47422, 16.1449}, {2.86095, 19.9929}}	{59.7255, 128.062}	inf	489.	613.678	{71., 1690.}	87.2483	0.401181	newly	574.	306.559	{35., 827.}	63.7778	0.557937	Reff	1.73539	2.76935	{2.46158, 2.85769}	59.5806	ar	70925.	92303.1	{88938., 93122.}	30.1419
40	R0	{2.24724, 13.7861}	{2.58189, 14.5857}	{{2.48103, 13.7658}, {2.59564, 14.6975}}	{14.8915, 5.83536}	inf	1281.	1476.92	{128., 4051.}	84.4543	0.434512	newly	906.	1013.	{98., 2712.}	80.7714	0.429234	Reff	2.19627	2.52089	{2.41794, 2.58983}	14.7805	ar	85257.	90294.3	{90122., 90609.}	5.90832
41	R0	{1.77635, 8.62406}	{2.65844, 14.3522}	{{2.5684, 13.7324}, {2.76203, 15.0558}}	{49.6572, 66.4206}	inf	634.	423.032	{34., 1217.}	64.511	0.543717	newly	682.	310.238	{24., 850.}	64.3416	0.651842	Reff	1.75212	2.63683	{2.54264, 2.7551}	50.4937	ar	72941.	91006.4	{89965., 92252.}	24.7672
42	R0	{1.80397, 5.4932}	{1.42785, 15.3884}	{{1.0158, 8.09734}, {1.85141, 19.7105}}	{24.7005, 180.136}	inf	36.	3.95082	{0., 8.}	92.2131	1.4821	newly	83.	2.32787	{0., 4.}	97.1953	2.13959	Reff	1.80242	1.456	{1.01579, 1.91367}	24.5516	ar	73232.	26187.1	{1., 68333.}	66.0299
43	R0	{2.83096, 16.7077}	{1.94617, 14.0718}	{{1.94617, 14.0718}, {1.94617, 14.0718}}	{31.2541, 15.7766}	inf	491.	188.51	{4., 449.}	65.1052	0.840946	newly	311.	119.078	{3., 300.}	65.1661	0.85681	Reff	2.80905	1.93862	{1.92659, 1.94582}	30.9865	ar	92740.	73514.4	{77402., 78496.}	20.7306
44	R0	{2.73678, 11.6021}	{1.47554, 16.8383}	{{1.22395, 12.1355}, {1.86902, 19.7105}}	{46.0847, 49.5197}	inf	128.	7.79167	{0., 20.}	93.9128	1.71073	newly	125.	4.20833	{0., 14.}	96.6333	2.11551	Reff	2.73147	1.51603	{1.22351, 1.86872}	44.4975	ar	92003.	33077.9	{2., 75947.}	64.0469
45	R0	{2.14235, 6.98601}	{1.37636, 17.9091}	{{1.22638, 13.6259}, {1.66638, 19.5412}}	{35.9073, 156.357}	inf	49.	5.13636	{1., 14.}	89.5176	1.24056	newly	74.	1.95455	{0., 4.}	97.3587	1.92626	Reff	2.14079	1.38673	{1.22637, 1.66596}	35.4591	ar	82974.	29148.4	{5., 67352.}	64.9924
46	R0	{2.23409, 9.21289}	{1.36213, 17.4512}	{{1.10133, 13.8428}, {1.66638, 19.5412}}	{39.0297, 90.974}	inf	58.	4.86957	{0., 12.}	91.6042	1.42	newly	78.	2.30435	{0., 6.}	97.0457	1.99686	Reff	2.23183	1.37713	{1.18977, 1.66623}	38.2958	ar	85163.	26838.3	{3., 67554.}	68.486
47	R0	{2.12258, 8.15265}	{1.52965, 14.5193}	{{1.0158, 8.09734}, {1.97176, 19.5412}}	{29.6175, 80.2178}	inf	73.	4.15686	{1., 8.}	94.3057	1.44621	newly	100.	1.94118	{0., 5.}	98.0588	2.12342	Reff	2.11978	1.6126	{1.18972, 1.97166}	26.842	ar	82690.	39293.8	{4., 79280.}	53.6502
48	R0	{2.71771, 10.4636}	{1.43084, 16.3939}	{{1.10133, 10.3394}, {1.67375, 19.7105}}	{47.3513, 60.8948}	inf	20.	2.91525	{0., 7.}	85.4237	1.07134	newly	23.	1.15254	{0., 3.}	94.9889	1.65443	Reff	2.71654	1.4427	{1.14093, 1.75642}	46.8919	ar	91716.	28577.7	{1., 67968.}	68.841
49	R0	{2.44369, 6.66477}	{1.51005, 12.0917}	{{1.0158, 8.09734}, {2.45321, 19.5412}}	{39.7644, 87.4552}	inf	7.	1.49	{0., 3.}	80.1429	1.00659	newly	14.	1.46	{0., 2.}	93.5714	1.5263	Reff	2.44335	1.51001	{1.01578, 2.45314}	39.76	ar	88827.	20966.6	{1., 79179.}	76.6757
50	R0	{2.77383, 17.9775}	{2.56288, 17.827}	{{2.53519, 17.5902}, {2.7811, 19.6712}}	{7.66272, 2.9994}	inf	1401.	562.756	{79., 980.}	62.725	0.582926	newly	799.	304.8	{37., 508.}	63.7881	0.592762	Reff	2.71353	2.52495	{2.48461, 2.53359}	7.09082	ar	92334.	89862.4	{89518., 90011.}	2.6794
